# The response to hepatitis a vaccine in children with JIA on immunosuppresive treatment

**DOI:** 10.1186/1546-0096-12-S1-P41

**Published:** 2014-09-17

**Authors:** Despoina Maritsi, Olga Vougiouka, George Vartzelis, Kyriaki Benetatou, Stavros Diamantopoulos, Maria Tsolia, Nick Spyridis

**Affiliations:** 1 2nd Department of Pediatrics, Medical Faculty, University of Athens, Athens, Greece; 2 Dept of Pediatrics, Medical Faculty, University of Thessaly, Larisa, Greece

## Introduction

Hepatitis A is an indolent disease with a varying clinical spectrum and consequences. Little is known about the immune response to hepatitis A virus (HAV) vaccination, in children with Juvenile Idiopathic Arthritis (JIA).

## Objectives

To assess the sero-protective efficacy and safety of immunization against hepatitis A in JIA patients on immunosuppressive treatment, not previously exposed to HAV, and compare this to healthy controls.

## Methods

Matched case control study including patients with JIA and controls. All subjects received two doses of inactivated anti-HAV at 0 and 6 months. Seroconversion rates and anti-HAV IgG antibodies were assessed 4 weeks after the first dose and at 4 and 52 weeks after the last dose. The protective level of IgG antibodies against HAV was set as antiHAV>20mIu/ml. Children were monitored for localized or systemic adverse events for three days and for disease relapse for three months post vaccination. Exclusion criteria were infection or disease flare concomitant or up to four weeks prior to immunization, recent use of steroids (<3m), known liver disease or immunodeficiency, and serum testing positive for HAV infection. Data were analyzed using SPSS 17.0.

## Results

Eighty-three patients with a mean age of 6.4 years completed the study; 21 had polyarticular JIA, 7 had psoriatic JIA, 16 had ERA and 39 had oligoarticular JIA. Twenty-one patients received anti-TNFa, 35 received methotrexate and 27 received both. The control group consisted of 76 healthy individuals (mean age 5.2 years). At four weeks after primary vaccination 48% of patients and 65% (P<0.01) of the controls attained seroprotection. Ninety-four percent of the patients and 99% (P=0.07) of the controls seroconverted four weeks after the second dose. Seroconversion rate was 91.5% for the patient group and 97% for the controls at 52 weeks post the second dose. Mean IgG concentration at 4 weeks was 45mIU/ml in the patient and 89mIU/ml in the control group (P=0.04) while it reached 118mIU/ml and 213mIU/ml after the second dose respectively (P=0.03). Subgroup analysis showed patients on biologics had better seroconversion rates compared to children on MTX or children on biologics+MTX. Vaccines were well tolerated. Mild adverse events were noticed in 7 patients and 8 controls. None of the patients developed a flare.

## Conclusion

Conclusion: Two doses of HAV vaccine are safe and effective in the majority of children with JIA, whereas a single dose seems inefficient. Systemic illness should not preclude from completion of the vaccination schedule. Seroconversion rates are lower in children whose treatment regime includes MTX.

## Disclosure of interest

None declared.

